# Beyond the gender binary: a survey of gender marginalization and social boundaries in Australian jazz and improvisation

**DOI:** 10.3389/fpsyg.2024.1412511

**Published:** 2024-07-22

**Authors:** Talisha Goh, Cat Hope, Louise Devenish, Margaret S. Barrett, Nicole Canham, Robert L. Burke, Clare Hall

**Affiliations:** ^1^Sir Zelman Cowen School of Music and Performance, Faculty of Arts, Monash University, Clayton, VIC, Australia; ^2^Faculty of Education, Monash University, Clayton, VIC, Australia

**Keywords:** jazz, improvisation, gender, music industry, marginalization, Australia, women, gender diverse

## Abstract

Jazz and improvisation have typically been associated with ideals of freedom and liberty; however, in practice these genres are known to be constrained by entrenched patterns of male domination and gender discrimination. Despite a large number of qualitative accounts evidencing persistent sexism and gender exclusion in the field, there exists a lack of empirical data to assess the scale of this phenomenon and substantiate smaller-scale research on gender inequality. In this paper, we employ boundary theory to report on a quantitative investigation of gender marginalization in jazz and improvisation in the Australian context, positioning gender as a symbolic boundary resulting in the social exclusion and marginalization of gender diverse individuals and women. An anonymous survey (n=124) was run over a period of five months, to explore the beliefs, attitudes, and experiences concerning gender, of people participating in Australian jazz and improvisation. A means comparison found that gender was a statistically significant indicator (*p* ≤0.05) on almost all measures, with gender diverse respondents significantly more likely to report the effects of marginalization than their (cisgender) counterparts. Additionally, the results indicated contrasting forms of musical engagement and marginalization across gender groups, with women perceiving exclusion to a lesser extent than gender diverse practitioners, and differing in their opinions regarding work opportunities. Lastly, a widespread but historically unspoken awareness of sexual harassment in the Australian jazz and improvisation industry was reported by all genders. This paper concludes with three recommendations for future research, policy and practice: 1. Specific targeted strategies are needed to address the manifold and complex forms of marginalization experienced by gender diverse people; 2. Heightened institutional visibility for marginalized groups is needed to change gendered narratives and highlight awareness of inequities; and 3. Enhanced safety measures are critically needed to address sexual harassment throughout the industry.

## 1 Introduction

Jazz and improvisation musical genres are characterized by a spirit of democracy and an ethic of freedom, involving individual and group negotiation of musical boundaries that seem to transcend factors such as race, gender, or social background (Tomlinson, 1991; Monson, 2007; Givan, 2020). However, as we discuss below, growing popular and scholarly discourse and literature recognizes that—far from its equitable and liberating ethos—gender marginalization remains an ongoing and widespread harmful issue for individuals practicing in these genres. This marginalization has been shaped by raced, gendered, and colonialist histories, leaving damaging legacies that can be observed in contemporary practices. For example, jazz and blues histories note that one of the few acceptable means for women’s participation in early jazz was as vocalists or “blues women.” This legacy continues in present-day gendering of musical roles, where men tend to comprise most jazz instrumentalists, with women primarily engaged as vocalists (Tucker, 2002; Davis, 2011; Kernodle, 2014; Wehr, 2016; Buscatto, 2022a,b; Yuhan, 2024). Competitive forms of virtuosity (such as jazz soloing) and associations between improvisation and masculinity serve as a means of gendered negotiation, and a space to assert a soloist’s musicianship and authority (Wong, 2016; Heyman, 2022; Johnson, 2022; Buscatto, 2022a,b; Nicols, 2024). Rustin-Paschal extended this premise in an account of Charles Mingus Jr. through examining how performances of “jazzmasculinity” shaped encounters with multiple figures in his life, explaining that such (black, cisgendered) masculinities were characterized by “expertise, discipline, and the mastery of self, others, and the music,” as well as “innovation, collaboration, expertise, and emotionality” and “an ability to nurture” (2017, p. 98, 128). Further, studies of contemporary musicians have emphasized that traits associated with the “jazzman” archetype continue to be performed by musicians of all genders and are still viewed as highly desirable within these communities today (Tucker, 2002; McAndrew and Widdop, 2021; Hall and Burke, 2022). Given its historical background and continuing “troubled relationship with gender” in contemporary practice, the ideals of justice and social equity that purport to distinguish jazz and improvisation from other artforms differ greatly from the cultural reality of these musical practices (Reddan et al., 2022, p. xiv).

This study examines how gender shapes the experiences and perceptions of individuals in Australian jazz and improvisation music industries. The gendered histories and cultures associated with these artforms have positioned masculinity as a symbolic boundary that continues to inform behaviors and attitudes toward individuals in contemporary practice, as well as participation and progression in jazz and improvisation music communities. Symbolic boundaries are determined by markers of socially constructed, in-group identification, that are used as a means of identity and cultural formation, and encompass attributes such as appearance, behaviors and attitudes, and patterns of consumption (Lamont and Molnár, 2002). Jazz and improvisation musicians, for example, might be identified through markers such as authoritative and powerful (cisgender masculine) physicality, extensive knowledge of musical catalogues, use of technical language, ideals of high/loud/fast virtuosity, as well as characteristics of emotional sensitivity, confidence, (hetero)sexual virility, and self-assured “cool” aloofness (Rustin-Paschal, 2017; Teichman, 2020; Boeyink, 2022; Devenish et al., 2024). Although symbolic boundaries are necessarily subjective and context-dependent, Lamont and Molnár argue that they are used to enforce real social boundaries, or the “objectified forms of social differences manifested in unequal access to and unequal distribution of resources (material and nonmaterial) and social opportunities” (2002, p. 168). Symbolic boundaries thus have different impacts upon individuals and groups in society; while they are important components of identity formation and belonging, they are also dependent upon distinctions between “in” and “out” groups, and can be used to enforce and uphold social boundaries through processes of inclusion and exclusion by the dominant or powerful group (Lamont, 2001). In jazz and improvisation, a sphere in which markers of musical credibility are conflated with social constructions of masculinity, internalized symbolic boundaries manifest in the preservation of a gendered social boundary through the exclusion and marginalization of individuals who do not fit into the hegemonic jazzmasculine ideal. Research of these social boundaries in the creative industries has tended to focus on issues of gender visibility and representation, marginalization due to stereotypes, and the gendered inequities within musical workplaces as a subset of the wider creative industries. As we discuss below, this exclusion and marginalization has been evidenced by a plethora of inquiries conducted by academic, government-affiliated, and industry bodies, that affirm a gendered symbolic boundary positioning women and gender diverse individuals as an out-group of Australian music and performing arts industries.

### 1.1 Representation

In Australian music and performing arts more broadly, imbalances in gender representation have been well documented. Numerous music industry reviews have found that women and gender diverse workers are underrepresented at the highest levels such as board membership or leadership positions, but overrepresented in lower support roles (Banks and Milestone, 2011; Browning, 2016; Cooper et al., 2017; Edmond, 2019). A similar picture has emerged in analyses of musical programming, commissions, and awards (Hope, 2022). In response, various organizations and festivals have taken initiatives to redress the lack of gender diversity, and new initiatives continue to improve diverse visibility (Browning, 2016; Cooper et al., 2017; Raine, 2019; McCormack, 2020; Tenth Muse Initiative, 2022). Although the effectiveness of these schemes in systemically addressing issues of underrepresentation is debated (Edmond, 2019), it is evident that there is high demand for representation; further, diverse representation across the broader Australian music and performing arts industry has great potential to impact jazz, improvisation, and popular music genres, which are highly influenced by commercial and market trends (Raine, 2019; McAndrew and Widdop, 2021). A number of qualitative artistic responses have also addressed this issue, such as saxophonist Cheryl Durongpisitkul’s work *A Pinky Promise* (including a movement entitled *Diversity Poster Girl*), performed by the Monash Art Ensemble in 2022. Although gender representation may appear to be a large step toward gender equity, it entails an accompanying increase in responsibility and emotional labor placed upon visible practitioners. Through the appearance of gender parity through representation, masking gender inequities under assumptions of meritocracy thus risks further marginalization of gender diverse and women practitioners in jazz and improvisation (Gill, 2014; Edmond, 2019). That is, without the structural and social changes to support it, representation alone cannot solve problems of jazzmasculine hegemony and gender exclusion.

### 1.2 Marginalization

Although there are attempts within the music industries to address issues of representation, some scholars argue that positive discrimination and other strategies to mitigate underrepresentation do little to change marginalizing behaviors that occur as a result of gendered social boundaries, and may unintentionally reinforce them (Gill, 2014; Edmond, 2019). In workplace studies, marginalization “represents the relegation of people (or groups of people) to a less powerful or included position within a society, and experiencing marginalization is a critical barrier to securing decent work” (Duffy et al., 2016, p. 132). A number of studies have focused on the effects of marginalization on individuals who do not fit into the jazzmasculine construct, finding that the gender identity of these outsider individuals tends to take precedence over intellectual and artistic contributions when superficial attempts at inclusion are made by practitioners holding positions of power (Wehr, 2016; Suzuki, 2022). Issues that result from this include tokenism, or the inclusion of “exceptional” women or gender diverse artists in male-dominated canons and repertoire on the condition of conforming to acceptable gender stereotypes such as jazzmasculinity (Tucker, 2002; Wehr, 2016), gendered expectations of labor within and beyond musical groups (Wehr, 2016; Buscatto, 2022a,b), and the devaluation of women and gender diverse musicians’ authority, capability, and musical contributions (particularly in the case of jazz vocalists, who tend to be women) (McAndrew and Widdop, 2021; Heyman, 2022; Buscatto, 2022a,b). Research conducted in the Australian scene supports the premise that the instrumental/vocal binary constitutes a gendered symbolic boundary among performers, resulting in gendered forms of marginalization (Hargreaves, 2013; Hargreaves and Forbes, 2022); for example, “vocalists…are often compared negatively to instrumentalists in terms of knowledge and application of jazz theory and musicianship”) (Istvandity, 2016, p. 75). Such marginalization serves to reinforce hierarchies of power, such as the objectification of jazzwomen as sex-objects or emotional laborers (Wehr, 2016), the valorization of “real” instrumental musicians, and equivalent assumptions of meritocracy within canons of jazz and improvisation (Whyton, 2010; Edmond, 2019; Buscatto, 2022a). Additional identities and factors—such as family or cultural origin, socio-economic status, disability status, or sexual orientation—also contribute to the individuals’ experiences of marginalization, often having a cumulative intersectional effect (Crenshaw, 1989).

### 1.3 Structural inequities

At a structural level, jazz and improvisation may be viewed as a particular microcosm of the wider creative and cultural industries, as it is characterized by a heavy reliance on formal and informal networks, indistinct boundaries between work and non-work, low remuneration (if any) for long hours of highly-skilled work, precarious modes of employment, and resulting cultures of competition (Gill, 2002). This has accumulated a particular set of inequities less often seen within traditional employment and organizational arrangements, with compounding effects on out-group exclusion and marginalization. For example, long and unpredictable hours are particularly unwelcoming to workers who are not men, as they are expected to take on the bulk of household and caring responsibilities (Banks and Milestone, 2011; Buscatto, 2022a). Further, the industry networks which are a vital means of obtaining job opportunities, tend to be homosocial and exclusionary, leading to the oft-cited “boys club” (Hennekam and Bennett, 2017; Buscatto, 2019). Concerningly, the culture of precarity and competitive individualism has also contributed toward the normalization of sexual violence and harassment within the music and creative industries, as victims risk career or reputational damage for speaking out (Hennekam and Bennett, 2017; Crabtree, 2020; MAPN Consulting and Support Act, 2022). Consequently, “serial perpetrators [of sexual violence and harassment] are often an open secret and are not always held to account” (MAPN Consulting and Support Act, 2022, p. 9). The invisible structures that govern the industries inhabited by musicians make it difficult for individuals to identify and voice instances and experiences of marginalization, leading to the reification of “unspeakable inequalities” within neoliberal cultural and creative spaces, in which “the new laboring subjectivity seems to demand a repudiation of structural inequalities” (Gill, 2014, p. 517).

### 1.4 Survey rationale

Given the challenges in identifying the specifics involved in gender marginalization and the ambiguities surrounding cultural and creative work, it is not surprising that rigorous quantitative investigations into the subject have been scarce. Due in large part to a lack of funding or support within institutional contexts, the few English-language academic articles dedicated to gender issues in jazz and improvisation have tended to be one-off unfunded investigations conducted by lone early-career women, many of whom had experienced gender marginalization as practitioners themselves (Canham et al., 2022). This sporadic interest in minimizing gender marginalization’s effects has resulted in difficulties maintaining long-term change in Australian music industries (Edmond, 2019). Although many studies of the Australian music and greater performing arts industries have explored gender marginalization in terms of descriptive statistics, inferential investigations are needed to determine the scope of the problem, identify the most pressing issues, and the groups that experience them. Our survey represents the only quantitative investigation of gender marginalization in Australian jazz and improvisation to date that we are aware of, and so presents a unique opportunity to bridge gaps between academic and industry investigations and inform policy in the area, which is often dependent upon statistical investigations. In turn, this will aid in galvanizing the industry from tacit awareness of the issue to meaningful action. This survey aimed to identify gendered perceptions of marginalization by taking an exploratory statistical approach to determine if factors of age, educational attainment, and ancestry might also be predictors. Although we view gender as a symbolic boundary influencing in and out-groups in jazz and improvisation, we considered it important to address the experiences of those who identify beyond the gender binary, who remain an under-researched group in wider workplace studies (McFadden and Crowley-Henry, 2016).

## 2 Materials and methods

This survey aimed to identify gendered perceptions of marginalization through an exploratory statistical approach, considering factors such as age, educational attainment, and ancestry. As there are many ways in which symbolic boundaries contribute to marginalization of out-group individuals, the survey design drew on intersectional feminist theories to address equality through democratic artistry and feminist musicology (McClary, 1991; Citron, 1993; Green, 1997). Marginalization impacts people differently according to the intersection of other structural inequalities, such as sexuality, race, ethnicity, class, age, and geography (Crenshaw, 1989; Hooks, 2015). A key component of the survey’s conceptualization, structure, and questions were the authors’ lived experiences and expertise as musicians and researchers in jazz, improvisation, and performing arts, particularly as most of the research team identified as members of the gender out-group. Additionally, the research team spanned a diverse range of career stages, means of artistic engagement, and industry experience, with specialist knowledge in fields such as gender studies, career counseling, musicology, sociology, narrative inquiry, music education, and artistic research methods. These knowledges were considered throughout the formulation of the survey; for example, the statements discussed in the Results section were shaped by Authors 2, 3, 5, and 6’s status as active practitioners in jazz, improvisation, new, and Western art music. As well as assessing its positionality, the team was mindful of the disruptive impacts of the COVID-19 pandemic on the Australian performing artists at the time of survey development, and questions were included where theory and literature pointed to a likelihood of possibility, such as in the case of sexual harassment (Hennekam and Bennett, 2017; Crabtree, 2020).

Using the Qualtrics platform, an initial pool of over 60 items was generated by the research team over a period of 4 months. This was reduced and condensed in line with survey research that recommends a maximum completion time of 20 min, the estimated average attention span for adults (Revilla and Ochoa, 2017). In total, 45 questions were used in the sector survey. The survey included 7 overarching sections, as follows:

Part 1: Explanatory Statement and Consent form (1 question)

Part 2: Tell us about yourself (8 questions)

Part 3: Education and training (7 questions)

Part 4: Practice (8 questions)

Part 5: Engagement with jazz and improvisation (13 questions)

Part 6: Motivations and gendered experiences (7 questions)

Part 7: Follow up and contact details (1 question)

Of the 7 sections, Parts 4–6 contain questions generated specifically for this survey with the remaining items covering demographic or background information. Feedback was sought from expert reviewers in the field to ensure the appropriateness of items and choice of tick-box answers, leading to several iterations of the questionnaire before it was sent out. Most questions (39 of 45) could be answered quickly, utilizing a multiple-choice, tick-box, drop-down selection, sliding-scale, or 5-point Likert scale format, and an open-ended “other” response was offered if the respondent did not identify with any of the categories suggested. Three questions required short 1–2 word typed responses, and three required larger open-ended responses. All questions were optional, meaning that respondents could proceed to the next question without answering. Under half (18) were governed by conditional logic, relying on the provision of a previous answer to show. For our purposes, and given that women and gender diverse musicians face challenges in accessing jazz and improvisation in a professional capacity (Buscatto, 2022a), the surveys were aimed at those who engage with the industry in any capacity, working from a model of inclusion rather than exclusion. This was reflected in the format and wording of our survey questions; for example, we asked participants to describe their artistic identities rather than limiting them to tick-box categories.

The survey was open for a period of 5 months from November 2021 to April 2022. Key industry bodies from around Australia, each with a large practitioner subscription database, were approached to distribute the survey URL in their mail-outs or newsletters. Musicians and workers known to the researchers were also contacted to spread the survey link within their networks, comprising a snowball sample. As an ethical consideration, a list of mental health and gender-specific support services was supplied to all respondents before commencing the survey in case they experienced any distress answering the questions. Participants were also given contact information for the research team to voice any questions or concerns, and informed that they could withdraw themselves and their data from the research at any time.

### 2.1 Data analysis

A series of mean comparisons were conducted for proportions at *p* ≤ 0.05 to examine whether factors of education level, age, ancestry, and gender played a role in motivations, participation, perceptions, and experiences in jazz and improvisation. In the Results section below, we report the significant findings from the survey questions relating to perceptions and experiences of marginalization.

## 3 Results

### 3.1 Demographic profile of final sample

Overall, a total of 184 responses were gathered; with 129 responses completed sufficiently for inclusion in the final analysis. Of these, five did not provide information about their gender identity. The following data analysis pertains to the final sample of 124 responses.

In alignment with our gender-inclusive practices and our own theoretical framework, respondents were offered an open-ended text box to describe their gender identity, rather than choosing from limiting and prescriptive categories (Barclay and Russell, 2017; Australia Council for the Arts, 2021; Lindqvist et al., 2021; Victoria State Government, 2021). The analysis (Table 1) divided self-described gender identities into 3 broad groups: Women, Men, and Non Binary/Gender Diverse (Cameron and Stinson, 2019). “Women” incorporated responses of “female,” “cis woman,” and “she/her,” while the “Men” categorization incorporated responses of “male,” “cis man,” and “he/him.” Due to the small sample size of the Non Binary and Gender Diverse groups, it was necessary to merge these groups for statistical analysis; this amalgamated group (referred to as “Gender Diverse” throughout this paper) included responses of “Female/Non Binary,” “Male/Non Binary,” “Transgender woman,” “Transgender man,” and “Genderfluid.” Of the 124 analyzed responses, 46.0% identified as women, 45.2% identified as men, and 8.9% identified as non-binary or gender diverse. While we acknowledge that our sample may not be representative of the gender makeup of the industry, we note there is no accurate information about that makeup currently available. Further, given that women and gender diverse practitioners tend to understate or underestimate their participation in the professional artistic workforce (Conor et al., 2015; Scharff, 2015; Buscatto, 2022a,b), we argue that incorporating all levels of participation (including self-identified “non-professionals”) provides a more accurate picture of gendered engagement in Australian jazz and improvisation.

**Table 1:** Demographic characteristics of respondents.

		Women *n* (% of total)	Men *n* (% of total)	Gender Diverse *n* (% of total)	Total *n* (%)
Gender identity		57 (46.0)	56 (45.2)	11 (8.9)	124 (100)
Age					
	18–29	20 (16.1)	10 (8.1)	4 (3.2)	34 (27.4)
	30–49	22 (17.7)	23 (18.5)	5 (4.0)	50 (40.3)
	50+	15 (12.1)	23 (18.5)	2 (1.6)	40 (32.3)
	Total	57 (46.0)	56(45.2)	11 (8.9)	124 (100)
Ancestry					
	Identifies Australian or British ancestry	45 (36.3)	46 (37.1)	9 (7.3)	100 (80.6)
	Does not identify Australian or British ancestry	12 (9.7)	10 (8.1)	2 (1.6)	24 (19.4)
	Total	57 (46.0)	56(45.2)	11 (8.9)	124 (100)
Educational attainment					
	High school	3 (2.4)	7 (5.6)	1 (0.8)	11 (8.8)
	Certificate, Diploma or other post-secondary certification	4 (3.2)	0 (0.0)	0 (0.0)	4 (3.2)
	Bachelor	12 (9.7)	7 (5.6)	2 (1.6)	21 (16.9)
	Bachelor (Honors)	4 (3.2)	6 (4.8)	0 (0.0)	19 (15.3)
	Graduate diploma or other graduate certification	7 (5.6)	11 (8.9)	1 (0.8)	10 (8.1)
	Masters	10 (8.1)	14 (11.3)	3 (2.4)	27 (21.8)
	Doctorate	12 (9.7)	10 (8.1)	3 (2.4)	25 (20.2)
	Not stated	5 (4.0)	1 (0.8)	1 (0.8)	7 (5.6)
	Total	57 (46.0)	56 (45.2)	11 (8.9)	124 (100)

**Figure 1:**
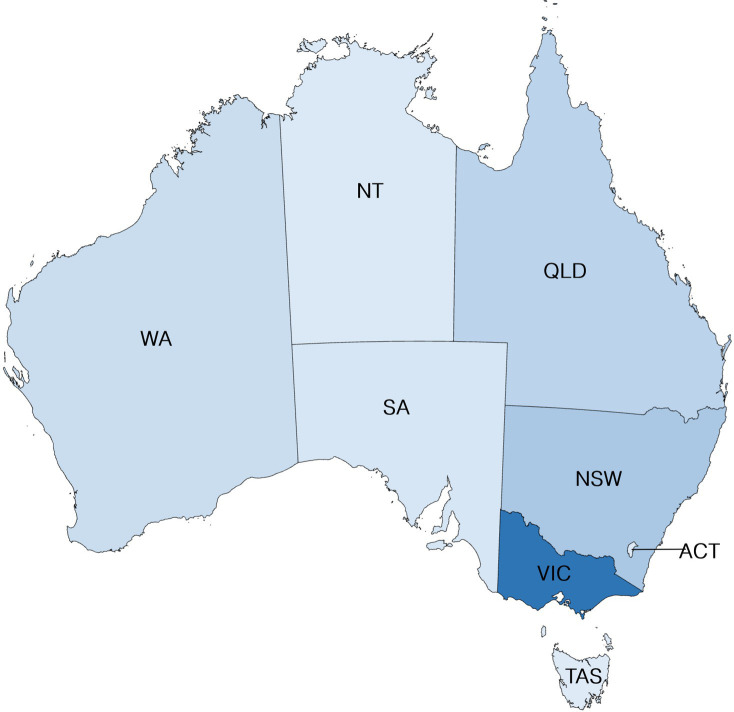
Geographical distribution of respondents.

Participants represented a range of ages, with just over a quarter (27.4%) from 18 to 29 years, 40.3% from 30 to 49 years, and almost a third (32.3%) over 50 years of age. Most were Australian born (84.7%), only spoke English (89.5%), and reported Australian or British ancestry (80.6%). Our sample also contained a high proportion of university-educated respondents, with postgraduate qualifications highly represented (Doctorate 20.2%; Masters 21.8%; Honors, Graduate Certificate, or Diploma 23.4%; Bachelor 16.9%). The remainder (12.1%) indicated they had completed high school with or without a further post-secondary certificate or diploma. By contrast, only half (51.6%) of respondents indicated that at least one of their parents had studied at university. Although respondents lived across Australia, many of our final sample were based in Victoria, despite considerable efforts to garner interest in the survey Australia wide through relevant contacts (Figure 1).

A broad range of musical practices within and around jazz and improvisation genres were indicated throughout the sample (Table 2). Respondents tended to have extensive musical experience (23.4% practicing for over 41 years, 25.8% 31–40 years, 21.8% 21–30 years; 25.8% 11–20 years; and 3.2% less than 10 years), specifically in jazz and improvisation (37.9% practicing more than 21 years since age 18, 23.4% 11–20 years, 17.7% 6–10 years; and 20.2% less than 5 years). More than half (51.6%) of respondents primarily practiced within jazz genres, encompassing styles such as bebop, modern jazz, and contemporary jazz (mean 42.7%, st. dev 29.5). Improvisational genres (including experimental and fusion) were the primary genre practiced by a quarter of respondents (26.6%, mean 28.1%, st. dev 24.3), alongside around a third based in other genres (including classical and pop) (30.6%, mean 29.2, st. dev 24.5).

**Table 2:** Basis of practice.

		Women *n* (% of total)	Men *n* (% of total)	Gender diverse *n* (% of total)	Total *n* (%)
Years of musical practice (any genre)					
	0–10	2 (1.6)	2 (1.6)	0 (0.0)	4 (3.2)
	11–20	19 (15.3)	10 (8.1)	3 (2.4)	32 (25.8)
	21–30	11 (8.9)	11 (8.9)	5 (4.0)	27 (21.8)
	31–40	15 (12.1)	15 (12.1)	2 (1.6)	32 (25.8)
	41+	10 (8.1)	18 (14.5)	1 (0.8)	29 (23.4)
	Total	57 (46.0)	56 (45.2)	11 (8.9)	124 (100)
Years in jazz and improvisation practice (since age 18)					
	5 or less	17 (13.8)	6 (4.9)	2 (1.6)	25 (20.2)
	6–10	10 (8.1)	8 (6.5)	4 (3.3)	22 (17.7)
	11–20	13 (10.6)	12 (9.8)	4 (3.3)	29 (23.4)
	21+	17 (13.8)	29 (23.6)	1 (0.8)	47 (37.9)
	No response		1 (0.8)		1 (0.8)
	Total	57 (46.0)	56 (45.2)	11 (8.9)	124 (100)
Primary genre practiced*					
	Jazz	28 (22.6)	33 (26.6)	3 (2.4)	64 (51.6)
	Improvisation	14 (11.3)	14 (11.3)	5 (4.0)	33 (26.6)
	Other genres	20 (16.1)	15 (12.0.1)	3 (2.4)	38 (30.6)
Artistic identity*					
	Jazz musician	22 (17.7)	27 (21.8)	5 (4.0)	54 (43.5)
	Improviser	22 (17.7)	25 (20.2)	1 (0.8)	48 (38.7)
	Performer (other)	17 (13.7)	10 (8.1)	3 (2.4)	30 (24.2)
	Instrumentalist	8 (6.4)	6 (4.8)	1 (0.8)	15 (12.1)
	Vocalist	9 (7.3)	2 (1.6)	0 (0.0)	11 (8.9)
	Composer/Songwriter/Arranger	13 (10.5)	12 (9.7)	2 (1.6)	27 (21.8)
	Other artistic identity	7 (5.6)	10 (8.1)	2 (1.6)	19 (15.3)
	No response	2 (1.6)	–	–	2 (1.6)

*Respondents were able to identify with one or more categories, hence totals do not equal 100%.

### 3.2 Experiences in the workplace

Perceptions of experiences were assessed using a 4-point Likert scale of agreement to a series of statements on working in jazz and improvisation. With regard to the way the respondents were treated within jazz and improvisation communities, gender played a significant factor in all cases. Men almost universally agreed that they felt respected (100%), valued (98.1%), equally considered in terms of artistic input (94.3%), adequately recognized (88.7%), and safe (98.1%) (Table 3). Women were also likely to somewhat or strongly agree with these statements, but to a lesser extent (feeling respected 93.9%; valued 89.8%, equally considered artistically 77.6%; recognized 81.6%; safe 91.8%). Gender diverse respondents were the least likely to agree to all statements, with only 60% feeling respected and 70% feeling valued by colleagues. Less than half felt their input was equally considered (40%), that they were adequately recognized (40%), or safe (44.4%) in their current workplaces. Concerningly, the gender diverse group was significantly more likely to report different experiences compared to the other two gender groups (*p* ≤ 0.001 in all cases).

**Table 3:** Experiences when working in ensembles, projects, and gigs in jazz and/or improvisation (*n* = 112).

	Somewhat/Strongly agree		
Currently, when working in ensembles, projects, and gigs in jazz and improvisation:	Women *n* = 49	Men *n* = 53	Gender diverse = 10
I am treated with respect	93.9%_a_	100.0%_a_	60.0%_b_
People of many different backgrounds are represented	38.8%_a_	58.5%_b_	30.0%_a,b_
I am valued by the ensembles and projects I participate in	89.8%_a,b_	98.1%_b_	70.0%_a_
My artistic input is considered equally with all other members	77.6%_a_	94.3%_b_	40.0%_c_
I receive adequate recognition for my input	81.6%_a_	88.7%_a_	40.0%_b_
People are treated equally and fairly	73.5%_a_	84.9%_a_	40.0%_b_
I feel safe	91.8%_a_	98.1%_a_	44.4%_b_

Differing lettered subscripts denote significant differences in gender identity groups selecting a given factor at the 0.05 level.

In addition to gender identity, age was found to have a significant impact on perceptions of working in the industry in general, with older respondents feeling more respected (100.0.%, *p* ≤ 0.05) and safe (100.0%, *p* ≤ 0.001) than 30–49 (respected 91.3%; safe 87.0%) and under-29 age groups (respected 90.0%; safe 86.2%). Overall, however, age was less likely to affect these perceptions than gender. Educational attainment and ancestry did not have an observable effect on these responses.

### 3.3 Perceptions of equality and diversity

As with the reported experiences working in the industry, gender identity was found to have a significant effect on overall perceptions of equality and diversity throughout the industry (Table 3). Over half of men surveyed perceived diverse representation in the projects they worked on, and less than half of women and gender diverse respondents did (men 58.5%; women 38.8%, gender diverse 30.0%; *p* ≤ 0.005). Similarly, male-identifying respondents tended to have a more favorable outlook on the way others were treated in the workplace, with 84.9% agreeing, compared to 73.5% of women and only 40% of gender diverse respondents (*p* ≤ 0.001). Age also had an impact on these perceptions; over-50s perceived more diverse representation (66.7%, *p* ≤ 0.001) and equality (86.1%, *p* ≤ 0.005) than 30–49 year olds (representation 43.5%; equality 67.4%) and under-29 s (representation 30.0%; equality 76.7%) age groups. No significant effects were found for education and ancestry.

### 3.4 Factors affecting opportunity

Following on from our previous findings on perceptions of diversity and equality, the factors that respondents thought most affected professional opportunities were also found to vary significantly by gender (Table 4). Respondents were asked to check three factors they felt most affected professional opportunities in jazz and improvisation from a range of possible demographic and social factors. Men and gender-diverse practitioners were more likely to look toward others as important determinants of opportunity, sharing the three most cited factors of networks, standards of expertise, and personal relationships. Women, on the other hand, recognized the role of outside roles and responsibilities, citing this factor over personal relationships, though this was not statistically significant (33.3% for outside roles versus 28.1% for relationships). Women were, however, significantly less likely to attribute socio-economic factors to professional opportunities compared to the other two groups (women 5.3%; gender diverse 27.3%; men 17.9%; *p* ≤ 0.05). Age, education, and ancestry were not found to have significant effects on these responses.

**Table 4:** Most important factors (in top three) impacting professional opportunities in jazz and improvisation (*n* = 124).

	Gender Identity of Participant		
What are the top three factors you think most affect professional opportunities in jazz and improvisation?	Women *n* = 57	Men *n* = 56	Gender diverse *n* = 11
Age	10.5%	10.7%	9.1%
Socio-economic background	5.3%_a_	17.9%_b_	27.3%_b_
Gender identity	15.8%	7.1%	18.2%
Ancestry / Cultural background	3.5%	3.6%	0.0%
Geographic background	5.3%	8.9%	9.1%
Sexuality	7.0%	1.8%	9.1%
Training institution attended	21.1%	30.4%	18.2%
Roles and responsibilities outside of jazz and improvisation	33.3%	21.4%	9.1%
Networks and associations with other people	59.6%_a,b_	69.6%_b_	36.4%_a_
Standards of expertise	50.9%	60.7%	36.4%
Personal relationships	28.1%	35.7%	36.4%
Other	12.3%	16.1%	9.1%

Differing lettered subscripts denote significant differences in gender identity groups selecting a given factor at the 0.05 level.

### 3.5 Inclusion, exclusion, sexual abuse, and safety

General experiences and overall perceptions of the industry were assessed using a 4-point agreement scale to a series of statements regarding inclusion, sexual abuse, and safety (Table 5). While most male-identifying respondents felt respected (94.3%), only three-quarters of women and gender diverse respondents did so (women 79.2%; gender diverse 75.0%). Similarly, men were more likely to feel included (92.5%) than women (62.5%) and gender diverse (50.0%) respondents. These findings were statistically significant (*p* ≤ 0.05 for respect; *p* ≤ 0.001 for inclusion).

**Table 5:** Experiences in participation in the jazz and/or improvisation (*n* = 109).

	Somewhat or Strongly agree		
When participating in jazz and improvisation in general:	Women (*n* = 48)	Men (*n* = 53)	Gender diverse (*n* = 8)
I am treated with respect	79.2%_a_	94.3%_b_	75.0%_a,b_
I feel uncomfortable because of my gender	43.8%_a_	18.9%_b_	75.0%_a_
I feel I am included	62.5%_a_	92.5%_b_	50.0%_a_
I experience sexual abuse or harassment	31.3%_a_	0.0%_b_	50.0%_a_
I have witnessed or heard of others experiencing sexual harassment	75.0%	64.2%	75.0%
I feel unsafe because of my gender	27.7%_a_	0.0%_b_	75.0%_c_
I feel unsafe because of factors beyond my gender	16.7%_a_	9.4%_a_	50.0%_b_
My sense of safety affects the jobs/gigs I choose to do	37.5%_a_	13.2%_b_	100.0%_c_

Differing lettered subscripts denote significant differences in gender identity groups selecting a given factor at the 0.05 level.

Experiencing or witnessing sexual harassment and abuse was common throughout the industry, with three quarters (75.0%) of women and gender diverse respondents, and more than half (64.2%) of men, seeing or hearing of instances. Alarmingly, almost a third of women (31.3%) and half of gender diverse (50.0%) respondents had experienced sexual abuse or harassment themselves, and this experience was not reported by any men (0%, *p* ≤ 0.001). Accompanying this finding, women and gender diverse groups were significantly more likely to feel unsafe because of their gender than men (women 27.7%; gender diverse 75.0%; men 0%; *p* ≤ 0.001). Gender diverse practitioners were also significantly more likely to feel unsafe due to factors beyond gender when compared to men and women (gender diverse 16.7%; women 27.7%; men 9.4%, *p* ≤ 0.005). Additionally, three quarters of gender diverse respondents felt uncomfortable because of their gender, compared to less than half of women and men (gender diverse 75.0%; women 43.8%; men 18.9%; *p* ≤ 0.005). Age, educational attainment, and ancestry were not found to have significant effects on these perceptions.

## 4 Discussion

This study explores the perceptions and experiences of individuals who participate in contemporary Australian jazz and improvisation, examining data along the axes of gender identity, age, educational attainment, and ancestry. In any social setting, symbolic boundaries are meaningful constructions in determining the collective interests, identity, and values of a group. Our study supports the premise that symbolic boundaries can “play an important role in the creation of inequality and exercise of power” through persistent marginalization of individuals who do not fit with in-group markers (Lamont et al., 2015, p. 850). This study provides statistical evidence for perceptions of marginalization on the basis of gender identity, demonstrating some ways in which gendered symbolic boundaries contribute to the exclusion of less powerful out-groups in jazz and improvisation. Further, the data analysis suggests that both gender out-groups experience and view marginalization in different ways, while their gender in-group colleagues report more respectful and inclusive perceptions of their contributions, and more optimistic views of many aspects of their experiences of the industry. For example, women were most likely to see outside roles and responsibilities as having an effect on professional opportunities, and gender diverse respondents were least likely to view networks in this way. The “double-status” of women, as part of the cisgender in-group and the non-male out-group, was reflected throughout their responses, as their reports of marginalization tended to fall somewhere between that of men (as a double in-group) and gender diverse respondents (a double out-group). Interestingly, no statistically significant differences were reported between ancestry groups or levels of educational attainment, with age being a predictor of perceptions of safety and respect to a small degree. Although a number of differences were found between the gender groups of this study, it should be noted that the same size of our survey (57 women, 56 men, and 11 gender diverse respondents) may limit the generalizability of the findings, and further large-scale investigations may expand on the recommendations below. Here, we contextualize three major findings of our survey, with recommendations for how these can inform future research policy and practice in Australian jazz and improvisation.

### 4.1 Recommendation 1: Deeper understandings and targeted support for gender diverse practitioners

Our findings point to the multifarious ways in which gender diverse individuals are marginalized throughout greater society. Our data suggests that symbolic markers of masculinity are particularly harmful to gender diverse people in Australian jazz and improvisation. When compared to (cis) women and men, gender diverse respondents had statistically significant differences across perceptions of respect, artistic valuation, and recognition when working in the field, and feelings of respect and inclusion from the industry in general, pointing to the complex and myriad ways that gender diverse individuals experience marginalization in everyday life. These findings align with studies of the broader workplace, which have found that gender diverse individuals face unique stressors beyond being openly gender non-conforming at work, including relational factors (e.g., visibility of gender minorities, inclusion in committees, and available networks), and practical factors (e.g., inclusive language use, availability of gender-neutral toilets and change-rooms, voice-related issues, and bullying and harassment) (Boncori et al., 2019). As part of the wider cultural and creative industries, jazz and improvisation workplaces are also complicated by fluid boundaries between work and non-work contexts as social and work spaces tend to overlap, which can lead to ambiguity regarding appropriate and inappropriate behavior in different contexts (Gill, 2002). Although our sample of gender diverse respondents was relatively small (*n* = 11), this data highlights a need for quantitative research specific to gender diverse groups in the cultural and creative industries more broadly, as they have received relatively little attention in existing research (Canham et al., 2022). Means of mitigating the specific forms of marginalization experienced by our respondents, which are situated among other various complex, compounding, and intersecting forms of oppression across many social contexts, would also benefit greatly from further in-depth investigations.

Beyond experiences and perceptions of exclusion, this study points to some of the effects of marginalization on the ways that gender diverse individuals engage with jazz and improvisation. For example, gender diverse individuals tended to cite socio-economic background as an important factor affecting job opportunities, downplaying the importance of standards of expertise and networks compared to cisgender women and men. This difference suggests that economic factors may remain a central limiting factor in gender diverse individuals’ engagement in jazz and improvisation; aligning with findings that show almost a third of LGBTQIA+ Australians earn less than a living wage and live below the poverty line (Hill et al., 2021, p. 26). Further, this research has found that due to social exclusion, gender diverse Australians may observe and use different strategies to participate musically, such as in the higher attribution of personal relationships in affecting job opportunities. This finding might suggest that inclusive communities are particularly important to gender diverse musicians, as knowledge of which opportunities are available, welcoming, and safe to them remains a primary concern.

Another more worrying effect of marginalization for gender diverse respondents was that less than half currently felt safe when participating in jazz and improvisation in general, with three-quarters specifying that they generally feel unsafe due to their gender, and half due to other factors. This finding reveals that, though there have been recent efforts to improve gender diverse safety and visibility in the industry, such as best-practice recommendations and the creation of guidelines for music venues, there is still much work to be done. Moreover, the very significant differences in perceptions and feelings of safety depending upon gender highlights the urgent need for different forms of education and awareness when it comes to the health and safety of all who work in the industry. We therefore support calls to enhance these safety measures, noting that contexts beyond formal venues and institutions should also be considered (Music Victoria, 2021; Baker, 2023; Goodwin and Vincent, 2023). As a group that experiences lower wellbeing outcomes than cisgender workers altogether (including gay and lesbian workers), the marginalization of gender diverse individuals in music and performing arts has considerable mental health implications that should be accounted for in future research (Hill et al., 2021; Donaghy and Perales, 2022). Intersections between gender and sexual identities, which fell beyond the scope of the current study, present a further site for investigation, as LGBTQIA+ identities have been presented as “a threat to heterosexuality” (Schilt and Westbrook, 2009, p. 442), or a disruption of the prevailing social boundaries that maintain gendered inequities (Madureira, 2012). In line with broader studies of LGBTQIA+ marginalization (Hill et al., 2021), we recommend that targeted strategies to address the specific forms of marginalization experienced by gender diverse groups are needed within the Australian jazz and improvisation industry to create positive change.

### 4.2 Recommendation 2: Make invisible groups visible

The data obtained from this survey indicate differing perceptions of equality, diversity and inclusion between gender and age groups, as older groups and men were more inclined to perceive jazz and improvisation as an equal and fair industry, where they felt respected and safe. These findings on inclusion align with prior organizational studies, which have found that those experiencing greater degrees of out-group marginalization are more likely to perceive the workplace as unfair and non inclusive, and that “majority group members are often unaware of, or take for granted, the privileges associated with their status” (Mor Barak et al., 1998, p. 98). While the older age group in the study did not constitute a majority, it should be noted that they tended to skew male, with over half of the 50+ age group identifying as men, and over half of the 18–29 age group identifying as women. We found that those less aware of—or more hesitant to acknowledge—existing inequities were older male respondents, and this is likely tied to their societal privilege and associated cascading advantage overall. This phenomenon may also be explained by the higher degree of in-group power within the industry, as older, male, workers are more likely to hold higher positions within the industry (Browning, 2016), and thus may hold a more privileged status due to seniority and the social hierarchies inherent in jazz and improvisation. Other possible explanations for ambivalence toward gender inequities in jazz and improvisation concern attrition within the older age group, as out-group workers leaving the field causes an increased homogeneity in the older age group (Edmond, 2019), or differences in perception due to age, as more dramatic differences in industry equity and diversity may be viewed across a longer time span. Within their specific working groups, however, women did tend to feel respected, valued, and recognized, such as in all-women jazz big bands or study programs. This may indicate the benefits of smaller-scale cultural change on the ground level, or collaboration strategies that reduce the effects of gender marginalization (Bennett et al., 2019; Edmond, 2019).

On the whole, while the findings show some ways in which marginalization differs between gender diverse people and women, they also highlight different ways in which each group engages with jazz and improvisation as a result of wider structures of marginalization. The data also suggest that gender diversity in the industry decreases as age increases, supporting prior findings that those belonging to out-groups are aware of their exclusion and marginalization, which may motivate them to leave the industry altogether (Browning, 2016; Cooper et al., 2017; Hope, 2022). In line with recommendations from the Australian popular music industry (Edmond, 2019), we recommend that promoting and celebrating less visible groups, through organizational initiatives or otherwise, will help disentangle jazz and improvisation from constructs of jazzmasculinity and its associated symbolic boundaries and spread awareness of various forms of marginalization to in-groups. Ultimately, such work is needed to transform existing gendered social boundaries, decrease attrition of marginalized groups, and diversify the industry for future generations.

### 4.3 Recommendation 3: Address safety and harassment issues

An alarming finding from the survey was the high prevalence and knowledge of sexual harassment in the industry. In male-dominated or androcentric spheres such as the cultural industries, sexual harassment is known to be widespread and tends to take on particularly insidious forms (Hennekam and Bennett, 2017; Bridges et al., 2020; Raj et al., 2020). The findings suggest that jazz and improvisation are no exception. Almost three quarters of respondents, regardless of gender, knew of others who had been sexually harassed or abused in the industry, and almost a third of women and half of gender diverse respondents reported experiencing abuse or harassment themselves. Contrastingly, zero instances of harassment were reported by surveyed men. The high rates of knowledge and experiences regarding sexual abuse and harassment among the survey cohort substantiates findings that such behavior is rife within the Australian music industries (Crabtree, 2020; Baker, 2023), and supports the premise that instances of harassment and harassers are widely known as an “open secret” (MAPN Consulting and Support Act, 2022, p. 9).

Recent studies of Australian music note that harassing behaviors include sexist comments, offensive remarks, sexual joking or innuendo in banter, unwanted sexual attention, and sexual assault (Crabtree, 2021), and perpetrators include audience members, peers, managers, or those in positions of power in the industry (Crabtree, 2021; Elmes and Knox, 2022, pp. 29–30). Additionally, a potentially worse prevalence of sexual harassment has been identified within the Australian contemporary music industry, with 72% of women, 39% of men, and 85% of “additional gender” practitioners experiencing at least one instance in their careers (*n* = 690) (MAPN Consulting and Support Act, 2022, pp. 31–34). Given the above knowledge and incidence of harassment, and the fact that many musicians (including respondents of this survey) are known to engage in multiple musical genres (Bartleet et al., 2020), it is unsurprising that the gender diverse respondents and women surveyed were significantly less likely to feel safe within the industry overall when compared to men. Additionally, as discussed in Recommendation 1, gender diverse respondents were particularly likely to feel uncomfortable and unsafe due to their gender, citing safety as a major concern in the jobs or projects they engaged in. In a musical landscape where only 15% of artists of any gender feel safe at work all the time (Elmes and Knox, 2022, p. 27) and 80% of victims do not report harassment (Baker, 2023), the data indicate that harassment remains a large component of gender marginalization. Consequently, we recommend that policy and practice initiatives to address safety and sexual harassment be strengthened. Such initiatives are paramount in mitigating gender-based inequities in the industry and improving the culture of jazz and improvisation (Support Act, 2023).

## 5 Conclusion

This study provides new theoretical and empirical insights on gender marginalization in jazz and improvisation. Like many musical genres, jazz and improvisation are rooted in historical constructions and practices of gender such that notions of masculinity and musicality have become intertwined. As a result, masculinity has become a symbolic marker in social and professional contexts centered upon jazz and improvisation in the present day, maintaining a set of very real inequities and the exclusion and marginalization of those who do not fit or conform to the jazzmasculine archetype (Lamont and Molnár, 2002; Rustin-Paschal, 2017). This survey of 124 individuals who participate in Australian jazz and improvisation examined perceptions and experiences of inclusion and exclusion, finding that gender diverse individuals, who are furthest from the jazzmasculine ideal, were significantly more likely to experience marginalization than (cisgender) women, who in turn were more likely to experiences these effects than (cisgender) men. Further, we found evidence that gender groups experience marginalization differently: while gender diverse individuals were significantly less likely than cisgender groups to perceive representation and fairness, as well as feel respected, recognized, and safe, women were more likely to attribute outside roles and responsibilities as a major factor impacting professional opportunities. Harassment and safety were of particular concern, with most respondents having knowledge or direct experience of harassment, corresponding to gendered feelings of safety and the limitation of professional engagement due to safety risk. The survey complements a plethora of descriptive and qualitative investigations in the literature, contributing a statistical viewpoint in the absence of large-scale quantitative investigations regarding gender marginalization within the Australian jazz and improvisation industry. As the spirit of jazz and improvisation is built upon the liberation and transformation of music and people, to ensure these practices can flourish in the 21st century, devotees and institutions must strive toward equity for all.

## 6 Limitations and direction for future research and practice

A number of limitations must be acknowledged in this research. Firstly, the sample may not be representative of the complete Australian jazz and improvisation sector; for example, although much of the existing literature points to a masculinized population, our sample consisted of almost equal numbers of cisgender women and men, with relatively high levels of education, mostly from backgrounds that can be described as “Anglo-Australian.” Although our sample size was small from a statistical standpoint, the jazz and improvisation industry in Australia is known to be a smaller part of the larger music industry in Australia compared to other countries, and the current study represents the largest-scale survey of gender marginalization in the industry that we are aware of. The true number and nature of individuals who participate in Australian jazz and improvisation, in a professional, non-professional, or other capacity remains unknown.

A second limitation concerns the nature of the research, as it was necessary to disclose the gendered nature of the survey to respondents. Due to this disclosure, the research may have attracted a sample that is more sympathetic or aware of gendered issues, or respondents may have been more inclined to respond according to social desirability bias (Krumpal, 2014). Informing respondents of response anonymization is hoped to have mitigated some of this effect (Krumpal, 2014).

Lastly, the landscape of practice is still recovering and changing after COVID-19 devastated much of the performing arts industry in Australia (Rusak et al., 2021; Crosby and McKenzie, 2022). Survey responses were collected from 2021 to 2022, capturing a particular point in time when parts of Australia were emerging from prolonged lockdowns, and the effects of venue restrictions and shutdowns were still very much felt by many respondents. As a result, many of the survey questions specified a time period of reference (either pre-pandemic, or at the time of answering the survey), and findings remain in line with much of the industry research that was conducted before the pandemic, as well as that which is emerging post-lockdowns.

Accounting for factors that have limited this research, the findings indicate a number of avenues warranting further investigation and recommendations for practice. Firstly, the data support the premise that there is an existing culture of gender inequity, marginalization, and sexual harassment in the Australian music industries (Crabtree, 2020), and that implications for safety are paramount. This is particularly important to those who rely on this as a main source of income, as safe conditions remain a priority for workers (Australian Human Rights Commission, 2013). Qualitative investigations to further explore the experiences of marginalization, particularly by gender diverse individuals and women in the industry, will be valuable to developing strategies to mitigate gender marginalization in the future. Secondly, a particular focus on gender diverse and LGBTQIA+ people operating in the cultural and creative spheres is a potential site of further investigation. As a highly marginalized population operating in a precarious and competitive industry, these accounts will be important to developing strategies to improve equity and diversity. Thirdly, although survey numbers were not sufficient to draw statistical conclusions concerning intersectional identities, the effects of multiple marginalized identities upon individuals operating in this industry will potentially reveal additional structural inequities that should be considered in future policies and practices.

## Data availability statement

The datasets presented in this article are not readily available because the dataset is restricted for use to the nominated researchers only and is not available to the public. Requests to access the datasets should be directed to Gender Diversity in Jazz and Improvisation research team, https://www.monash.edu/arts/music-performance/genderjazz.

## Ethics statement

The studies involving humans were approved by Monash University Human Research Ethics Committee (MUHREC). The studies were conducted in accordance with the local legislation and institutional requirements. The participants provided their written informed consent to participate in this study.

## Author contributions

TG: Conceptualization, Data curation, Formal analysis, Investigation, Methodology, Project administration, Writing – original draft, Writing – review & editing, Supervision, Visualization. CaH: Conceptualization, Formal analysis, Funding acquisition, Investigation, Methodology, Supervision, Writing – review & editing. LD: Conceptualization, Formal analysis, Funding acquisition, Investigation, Methodology, Supervision, Writing – review & editing. MB: Conceptualization, Formal analysis, Funding acquisition, Investigation, Methodology, Supervision, Writing – review & editing. NC: Conceptualization, Formal analysis, Funding acquisition, Investigation, Methodology, Supervision, Writing – review & editing. RB: Conceptualization, Formal analysis, Funding acquisition, Investigation, Methodology, Supervision, Writing – review & editing. ClH: Conceptualization, Formal analysis, Funding acquisition, Investigation, Methodology, Supervision, Writing – review & editing.
